# Duodenal compressions from non-superior mesentery artery structures in the same patient causing superior mesentery artery-like syndrome: a rare case report

**DOI:** 10.1093/bjrcr/uaae037

**Published:** 2024-10-18

**Authors:** Hajar Andour, Amine Naggar, Wafae Khatibi, Hiba Zahi, Kaoutar Imrani, Nabil Moatassim Billah, Itimade Nassar

**Affiliations:** Abdominal Radiology Department, Ibn-Sina Hospital, Rabat, Morocco; Abdominal Radiology Department, Ibn-Sina Hospital, Rabat, Morocco; Abdominal Radiology Department, Ibn-Sina Hospital, Rabat, Morocco; Abdominal Radiology Department, Ibn-Sina Hospital, Rabat, Morocco; Abdominal Radiology Department, Ibn-Sina Hospital, Rabat, Morocco; Abdominal Radiology Department, Ibn-Sina Hospital, Rabat, Morocco; Abdominal Radiology Department, Ibn-Sina Hospital, Rabat, Morocco

**Keywords:** SMA syndrome, SMA-like syndrome, abdominal imaging, CT scan

## Abstract

Superior mesentery artery (SMA)-like syndrome is an increasingly used term to describe vascular compression of the third duodenal portion between structures other than the superior mesenteric artery and aorta. Although rare, this clinical condition is as serious as true SMA syndrome and requires similar management. However, the diagnostic criteria are not well established yet and require a case-by-case analysis, including a review of various clinical symptoms, especially evolving ones, as well as radiological imaging and effectiveness of conservative therapeutic manoeuvres.

The presented case involves a double vascular compression in a 50-year-old woman with no medical history, one of which is between 2 venous structures. The patient had been experiencing recurrent abdominal pain, vomiting, and distension for a long time. Laboratory tests were normal, and gastroesophageal endoscopy revealed Barrett’s oesophagus. CT-enterography revealed 2 duodenal vascular compressions. Dietary measures were initiated with close follow-up. To the best of our knowledge, this is the first reported case in the world literature and adds to the existing body of SMA-like syndromes.

## Introduction

Superior mesentery artery (SMA)-like syndrome is a recently identified but lesser-known condition that represents a variant of true SMA syndrome, being equally serious. It is characterized by duodenal vascular compression between structures other than the aorta and an abdominal vessel other than the superior mesenteric artery, typically another artery but occasionally a vein. Both the true syndrome and SMA-like syndrome share the same clinical presentation and predispose to high morbidity and mortality if not managed correctly. Limited literature reports describe SMA-like syndrome with different involved vessels, some of which are associated with recently discovered venous variants of mesenteric drainage.^1^ This manuscript describes the first observation in the literature of double vascular compression of the third duodenal segment at two levels, compatible with SMA-like syndrome. The first compression involves two veins: the inferior vena cava (IVC) and the right colic vein. The second compression occurs between the aorta and the inferior mesenteric vein, immediately following the first one.

## Case report

A 50-year-old woman presented with recurrent chronic epigastric pain for 7 years, which had been treated symptomatically following normal initial endoscopy and abdominal CT scan. Five years ago, due to unexplained persistent epigastric pain, another gastro-oesophageal endoscopy was performed, revealing biopsy results suggestive of Barrett’s oesophagus upon anatomopathological examination. Treatment with dietary and lifestyle modifications, including small non-acidic meals, was initiated. There was a transient improvement; however, subsequent recurrence of epigastric pain with bile reflux, vomiting, and an unintended weight loss led to further investigation.

**Figure 1.**
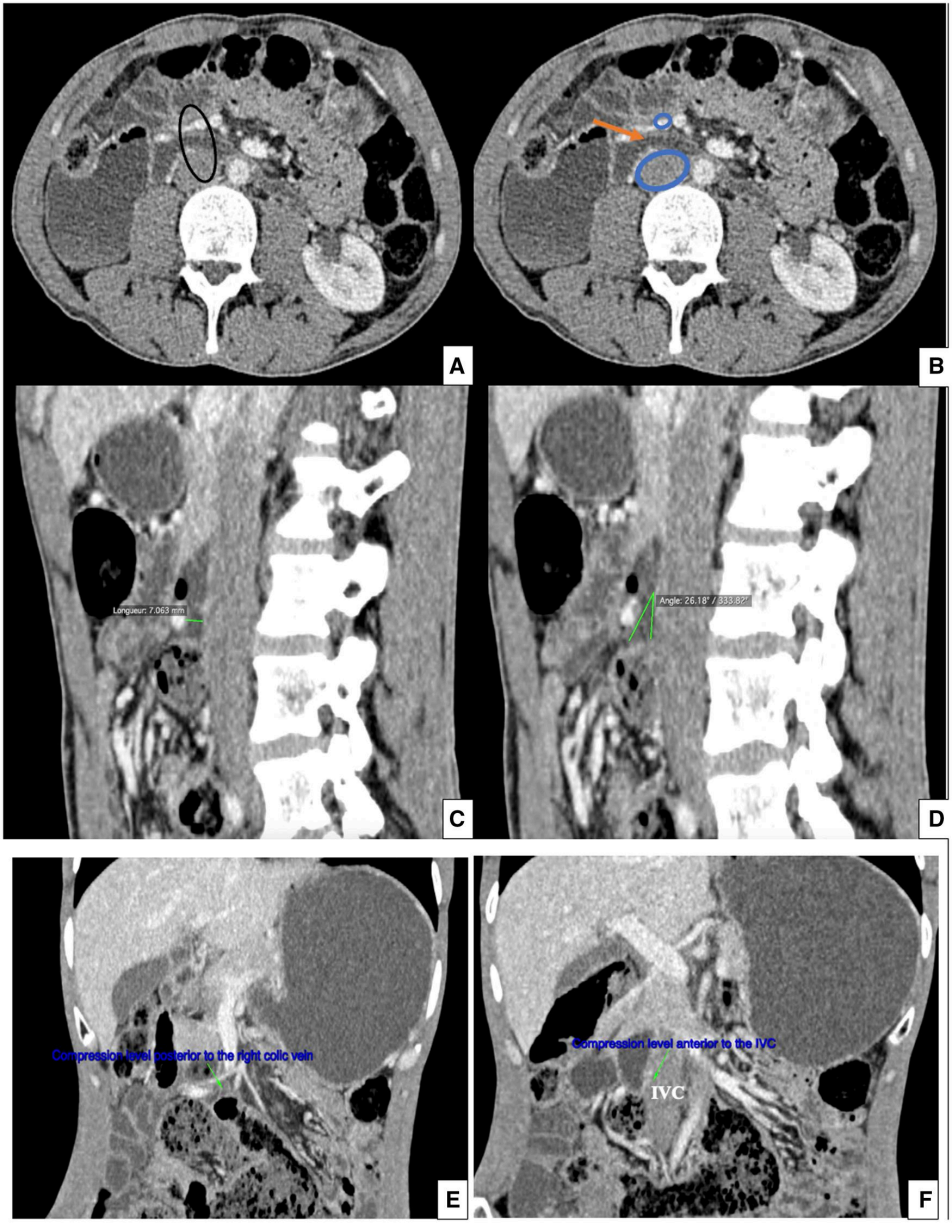
Axial (A, B), sagittal (C, D), and coronal (E, F) CT-enterography show the first level 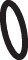 of compression of the third duodenal segment 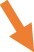 between the inferior vena cava 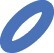 and the right colic vein. 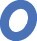 Note the angle and the distance.

**Figure 2.**
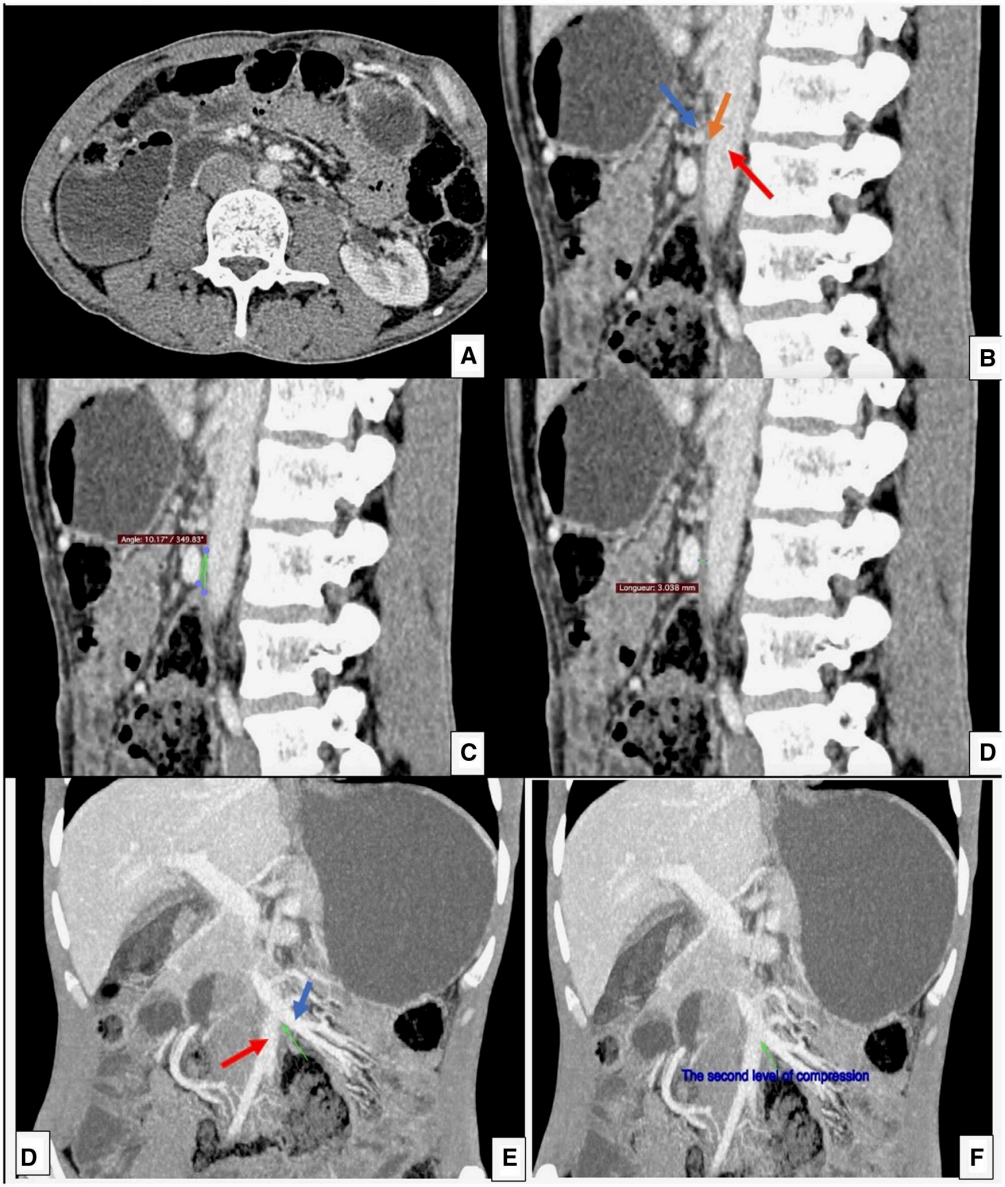
Axial (A), sagittal (B–D), and coronal (E, F) CT-enterography show the second level of compression of the third duodenal segment (middle orange arrow) between abdominal aorta (posterior red arrow) and the inferior mesenteric vein (anterior blue arrow). At this level, the compression is more marked with a narrowed angle.

Laboratory tests were normal. A CT-enterography was performed, revealing a liquid-gaseous distension of the first and second segments of duodenum, with abrupt tapering of the third segment at two points. The first point of compression was found between the IVC and the right colic vein, with an inter-venous distance of 7 mm and an angle of 26° (Figure 1). The second more pronounced point of compression was located adjacent to the first, between the abdominal aorta and the inferior mesenteric vein, with an aorto-venous distance of 3 mm and a sharper angle of 10° (Figure 2). Hygienic and dietary measures were initiated with close follow-up and monitoring to assess improvement and determine whether surgery was necessary. Three months later, the patient reported improvement in her clinical symptoms and maintained good adherence to her lifestyle.

## Discussion

SMA-like syndrome is a recently coined term used to describe the compression of the third duodenal segment by vascular structures other than the SMA and aorta. Grouped under the heterogeneous group of “vascular compression syndromes,” this entity could be considered a variant of the SMA syndrome or “chronic duodenal ileus” as previously described by Rokitansky and Wilkie. However, it does not meet the strict diagnostic criteria of SMA syndrome.^2^^,^^3^

Our patient presents a unique case, the first reported instance of a double duodenal compression, by 2 adjacent SMA-like syndromes.

The incidence of SMA-like syndrome is still unknown, but is likely lower than that of Wilkie’s syndrome—the original SMA syndrome, which has been reported to be less than 0.34% with two-thirds of patients aged between 10 and 39 years, predominantly females, although it is believed to be underestimated.^4^

The pathophysiology of SMA-like syndrome is similar to that of Wilkie’s syndrome. The acute downward angle of the SMA from the aorta leaves the third portion of the duodenum, suspended by the ligament of Treitz, vulnerable to compression between the artery anteriorly and the aorta and the vertebral column posteriorly. Consequently, various conditions that narrow the angle or shorten the distance between these structures predispose to SMA syndrome. The conditions could be congenital or acquired, including a shorter or hypertrophic Treitz ligament, significant weight loss, hypermetabolic states such as trauma and burns, dietary conditions like anorexia nervosa and malabsorptive diseases, as well as conditions associated with AIDS, cancer, and paraplegia. The syndrome may also occur following surgical correction of scoliosis, in the presence of peritoneal adhesions, Ladd’s bands, abdominal aortic aneurysm, mesenteric root neoplasms, and lumbar hyperlordosis.^5^

In the cases of a SMA-like syndrome, the majority of available reports suggest that there should be an associated pathological condition of the duodenum when its vascular compression is caused by structures other than the SMA and the aorta. This duodenal dilatation in typically part of a megaduodenum, which may occur in various conditions such as scleroderma, systemic sclerosis, dermatomyositis, diabetes, amyloidosis, and chronic idiopathic intestinal pseudo-obstruction, all leading to a diminished intestinal muscle tone and decreased peristalsis.^4^^,^^5^

Our patient, however, had none of these medical pathologies during a long-term follow-up. The Barrett’s oesophagus she developed years after the initial symptoms was mainly a consequence of recurrent vomiting and bile reflux caused by the syndrome.

Moreover, she is the first reported case of two vascular compression levels, with 1 level being between 2 venous structures. Vascular compression by veins is generally rare, as the venous wall is thinner. In this case, we strongly believe that the first compression was favoured by the adjacent one downstream. The latter had a very narrow angle and short distance, which predisposed the upstream duodenal segment to be distended and subsequently pinched.

The recorded distance and angle values clearly confirm this observation, with the values of the second level being much lower than the first one. Furthermore, it is noteworthy that these values are lower in venous vascular compression without associated duodenal pathology than with arterial vascular compression.

On the other side, even in the case of an arterial compression, it currently stated that in the absence of the required measurements of distance and aortomesenteric angle, and in front of unexplained symptoms, higher threshold measures could be implicated. Comparing these situations to previous imaging examinations and values is of great interest, as it will help in highlighting the acute decrease in these values as well as variations in the amount of retroperitoneal fat pad. Moreover, the severe and acute decrease of retroperitoneal fat pad has been correlated with worsening of symptoms.^6^^,^^7^

An anatomic variant of the superior mesenteric vein has been linked to SMA-like syndrome involving a dual venous trunk where one trunk may lie dorsally to the artery, between the SMA and the duodenum. Studies of mesenteric veins variations are limited. Tanakori Sakaguchi et al. (2009) classified superior mesenteric vein (SMV) variations into 3 groups: a single trunk (I), a double trunk joining the caudal side of the splenoportal confluence (IIm), and a double trunk where the left vein joins the splenic vein (IIp). More recently, Shiqi Guo et al. (2023) observed the double SMV variant, classifying it into type I (common trunk formation) and type II (no common trunk), with further subtypes based on colonic vein confluence.^8^^,^^9^

The management of both SMA and SMA-like syndromes should be approached as a serious multidisciplinary goal, particularly in complicated acute cases requiring the expertise of anaesthesiologists, radiologists, general surgeons, and gastroenterologists. This management typically begins with conservative nutritional support, including multiple small feedings and both oral and parenteral hyperalimentation to increase retroperitoneal fat pad until surgical intervention is indicated, which usually involves gastrojejunostomy or duodenojejunostomy. These therapeutic interventions are expected to be effective regardless of the nature of the vascular duodenal compression, including venous compression and its variants. Positional manoeuvres, such as postprandial lateral decubitus and procubitus, can be helpful in relieving pain positions and are used for both diagnostic and therapeutic purposes.^10^

## Conclusion

The diagnosis of SMA syndrome is based on well-established criteria. However, cases not meeting those criteria but where a vascular duodenal compression is identified are increasingly recognized as “SMA-like syndromes.” These are not limited to compression between an artery and a vein, but can also involve 2 venous structures. Understanding vascular venous anatomy and its variants is crucial in mastering these syndromes.

## Learning points

- SMA-like syndrome refers to the compression of the third segment of the duodenum by vascular structures other than the SMA and aorta, classified under vascular compression syndromes.
- Similar to SMA syndrome, SMA-like syndrome occurs due to the acute downward angle between 2 vascular structures, which may include venous ones, leading to duodenal compression.
- Factors such as congenital anomalies, significant weight loss, and certain diseases can increase the risk of developing the syndrome.
- Linking evolving clinical symptomatology to associated factors and their consequences on vascular anatomy and duodenal compression is important for establishing the diagnosis.
- Initial treatment typically includes conservative nutritional support and positional manoeuvres, progressing to surgical interventions like gastrojejunostomy or duodenojejunostomy when necessary.
